# A Rare Case of Severe Diabetic Ketoacidosis in a Patient With Metastatic Renal Cell Carcinoma Being Treated With Nivolumab

**DOI:** 10.7759/cureus.29537

**Published:** 2022-09-24

**Authors:** Talal Bazzi, Eisha Gupta, Ayman Mohamed, Megha Vashi

**Affiliations:** 1 Internal Medicine, Ascension St. John Hospital, Detroit, USA; 2 Internal medicine, Ascension St. John Hospital, Detroit, USA

**Keywords:** nivolumab, diabetic ketoacidosis, cancer treatment, cancer treatment side effects, autoimmune diabetes, metastatic renal cell carcinoma, nivolumab-related adverse events

## Abstract

Immune checkpoint inhibitors are becoming of more use as clinicians are prescribing them for patients with different malignancies. As their use continues to increase, clinicians must be aware of the side effects, which are autoimmune in nature. Autoimmune diabetes has been described in the past while patients were being treated with programmed cell death protein 1 (PD-1) inhibitors, but it usually occurs after the patient's fourth or fifth cycle. In this case presentation, we describe a patient with no history of type 1 or 2 diabetes presenting to the emergency department with severe diabetic ketoacidosis. At the time of presentation, he was on his 22nd cycle of nivolumab for metastatic renal cell carcinoma. The patient was eventually treated successfully, but upon discharge, he was prescribed a large dose of insulin regimen to control his blood sugar levels at home. We attributed his new diagnosis of insulin-dependent diabetes to the PD-1 inhibitor nivolumab.

## Introduction

There has been a great deal of advancement in cancer immunotherapy in multiple different malignancies, which have been refractory to standard chemotherapy in the past [1]. As clinicians begin to use more immune checkpoint inhibitors, immune-related adverse events continue to be on the rise [1]. It is estimated that immune-related adverse events in patients who are being treated with immune checkpoint inhibitors are roughly 10-20% and these events can be relatively serious with high morbidity and mortality [1]. There have been a few case reports introduced in the literature of patients who are on immune checkpoint inhibitors and presenting with diabetic ketoacidosis (DKA) and new onset insulin-requiring diabetes after the patient's fourth or fifth cycle of the drug; this occurs in less than 1% of patients who are on these drugs [2]. We present a 48-year-old patient with metastatic renal cell carcinoma on his 22nd cycle of nivolumab, with no history of diabetes, presenting to the emergency department with severe DKA.

## Case presentation

A 48-year-old male with a past medical history of metastatic renal cell carcinoma on immunotherapy presented to the hospital with a chief complaint of nausea, vomiting, and malaise. The onset of symptoms was roughly five days prior to presentation. The patient also complained of polyuria and polydipsia. He denied any fevers, chills, shortness of breath, chest pain, hematemesis, or hemoptysis. The patient’s vital signs were significant for sinus tachycardia with a rate of 110-120 beats per minute, and the remainder of the vital signs were within normal limits. A physical exam revealed dry mucous membranes but was otherwise unremarkable. Laboratory studies were obtained, and venous blood gas revealed a pH of 7.03 with normal partial pressure of carbon dioxide (pCO2) and partial pressure of oxygen (pO2) levels indicating severe metabolic acidosis. Complete blood count was significant for leukocytosis at 19.41 K/mcL and a hemoglobin level of 17.9 gm/dL with a baseline of 12-13 gm/dL. Most notably, the patient was hyperglycemic at 1190 mg/dL with a bicarbonate level of 6 mmol/L and his anion gap was markedly widened at 41. The patient also had an acute renal injury with a creatinine of 2.48 mg/dL, elevated from a baseline of 1.3-1.4 mg/dL. There was a type B lactic acidosis of 7.2 mmol/L. Urinalysis was positive for both glucose and ketones.

Given the anion gap metabolic acidosis, hyperglycemia, and ketosis, the patient did appear to be in severe DKA. The patient was treated for DKA with intravenous short-acting insulin, intravenous fluid resuscitation, and electrolyte repletion with frequent monitoring of his blood glucose levels and basic metabolic panels. The patient did require significant titration of the insulin drip with more than 100 units of short-acting insulin administered through the insulin drip and injection combined. Later, the patient's DKA did resolve but the patient did require a basal-bolus insulin regimen, which consisted of 30 units of long-acting insulin at bedtime with 10 units of short-acting insulin with meals and this was his home regimen as well.

Interestingly enough, the patient neither had a personal or family history of diabetes nor had he been on any diabetic medications in the past. The only medication the patient was taking was nivolumab, a programmed cell death protein 1 (PD-1) immune checkpoint inhibitor for his metastatic renal cell cancer, which he had been on for approximately 10 months. The patient was currently on his 22nd cycle of immunotherapy and has been receiving his infusion every two weeks. The last time he received his infusion was three days prior to the presentation.

Upon chart review, the patient had a glycosylated hemoglobin (HbA1c) level of 4.8% one year prior and the HbA1c level on this admission was 6.5%, which only just crosses the threshold to be diagnostic for diabetes. To evaluate for type 1 versus type 2 diabetes, a C-peptide level was obtained, which was markedly low at 0.5 ng/mL, indicating poor insulin reserve. Pancreatic insufficiency due to pancreatitis in the setting of metastatic renal cell carcinoma was also considered; however, recent abdominal imaging was negative for pancreatic metastasis, and amylase and lipase levels were within normal limits, making this etiology less likely. This picture appeared to be more consistent with autoimmune type 1 diabetes mellitus. Given that the patient was receiving treatment with immune checkpoint inhibitors, it was believed that the patient’s immunotherapy did induce fulminant type 1 diabetes mellitus resulting in severe DKA.

## Discussion

There are roughly 65,000 new cases of renal cell carcinoma diagnosed each year in the United States, with an estimated 15,000 deaths [3]. Nivolumab is a PD-1 immune checkpoint inhibitor antibody that selectively blocks the interaction between PD-1 and its ligands PD-L1 and PD-L2; a mechanism that normally leads to downregulation of cellular immune response and this allows enhancement of T cell function allowing for more anti-tumor activity [1]. In 2015, the US Food and Drug Administration approved nivolumab for patients with advanced renal cell carcinoma [4].

As one can predict, the side effects of immune checkpoint inhibitors are usually autoimmune in nature [2]. The side effects of these medications include, but are not limited to, pneumonitis, colitis, and endocrinopathies such as thyroiditis and hypophysitis [2]. Autoimmune diabetes mellitus can also occur and it happens when auto-reactive T cells begin to destroy pancreatic beta cells that produce insulin [4]. Interestingly enough, however, if nivolumab were to cause autoimmune diabetes, it usually occurs early on; roughly in the first five cycles of therapy [5]. In this patient, he received a total of 22 cycles of nivolumab before presenting with DKA. This is another reason why this case is rare, and the onset of DKA this late in the patient’s cycle regimen is relatively uncommon.

Predicting who will develop type 1 diabetes or go into DKA in patients on anti-PD-1 therapy is a challenge [5]. A study done by Magis et al. tried to determine whether or not looking at glucose levels before and after anti-PD-1 therapy can help determine whether one is at increased risk of type 1 diabetes [5]. Magis et al. advise against monitoring blood glucose in patients on immunotherapy because during and after anti-PD-1 treatment, all cases of type 1 diabetes occur in patients who had normoglycemia rather than hyperglycemia with normoglycemia prior to their DKA occurrence [5].

Another study done by Yun et al. looked into checking/trending HbA1c levels and whether or not that can help determine if one is at increased risk for developing type 1 diabetes once he or she started anti-PD-1 therapy [6]. The study concluded that trending HbA1c likely has no value in approximating the incidence of fulminant diabetes [4]. For example, in one of their patients, HbA1c about 2.5 months prior was 5.6% and then 9.3% at the time of DKA. Interestingly enough, however, a study done by Stamatouli et al. explained and showed that patients with a more elevated HbA1c at the time of their diagnosis of DKA while being on anti-PD-1 therapy had potentially more significant hyperglycemia as opposed to others with lower HbA1c [7].

For most immune-related adverse events, the mainstay therapy for treatment is glucocorticoids [5]. However, in the case of type 1 diabetes and/or DKA, the mainstay course of treatment is insulin [6]. Glucocorticoids are not usually used for the management of immunotherapy-induced type 1 diabetes as their common side effect is hyperglycemia. Patients who were diagnosed with immunotherapy-induced type 1 diabetes were to be continued on home insulin, but the resumption of the anti-PD-1 therapy usually ended shortly after, as patients had progression of disease necessitating a different choice in therapy [6].

Patients who develop DKA and are on anti-PD-1 therapy likely will reach a diagnosis of autoimmune diabetes, especially if they do not have a history of diabetes or risk factors such as obesity that makes them prone to diabetes [6]. As the use of anti-PD-1 therapy expands, clinicians have to be more alert to the possibility of their patients developing autoimmune diabetes [7]. Also, more studies have to be completed on ways clinicians can screen patients on immunotherapy who are at increased risk for developing autoimmune-related adverse events such as autoimmune diabetes.

## Conclusions

PD-1 immune checkpoint inhibitors work by causing downregulation of one’s own cellular immune response and that further allows for enhanced T cell function to have anti-tumor activity. In this case, this patient was being treated with nivolumab for metastatic renal cell carcinoma and presented with severe DKA. He was discharged home on insulin therapy for likely a new diagnosis of autoimmune type 1 diabetes. As the widespread use of immunotherapy continues to expand, clinicians have to consider screening for new onset autoimmune conditions. Should providers begin screening for diabetes with a hemoglobin A1C level in patients who are on immunotherapy? Future studies need more correlative data regarding screening those on immunotherapy with a high risk for the development of autoimmune diabetes.
